# Supplementary material for: Dynamic allostery in substrate binding by human thymidylate synthase
Source: eLife. 2022 Oct 6;11:e79915. doi: 10.7554/eLife.79915 (PMC9536839; doi:10.7554/eLife.79915)
Supplement: Supplementary file 1. — (a) values from global fit with pb≅1.3%, kex,ab=240s-1 (b) population and exchange rate shown for slower of two processes. Unlike the B↔A↔C model, which gives very similar pb and kex,ab for individual fits of these probes, the A↔B↔C model yields heterogeneous values, primarily for the exchange rate (c) χred2 is calculated as ∑iresidualierrori2numdatapoints-numfitparam [file elife-79915-supp1.docx]

|  | 2-state | B↔A↔C^a^ | A↔B↔C^b^ |
| --- | --- | --- | --- |
| V79 | $\chi_{red}^{2}=0.95$^c^ | $\chi_{red}^{2}=0.83$ | $\chi_{red}^{2}=0.76$  $k_{ex,ab}=200 s^{-1}$  $p_{b}=0.5\%$ |
| L101 | $\chi_{red}^{2}=0.74$ | $\chi_{red}^{2}=0.52$ | $\chi_{red}^{2}=0.58$  $k_{ex,bc}=2500 s^{-1}$  $p_{c}=1.2\%$ |
| L121 | $\chi_{red}^{2}=2.22$ | $\chi_{red}^{2}=1.83$ | $\chi_{red}^{2}=1.70$  $k_{ex,bc}=5400 s^{-1}$  $p_{c}=18.3\%$ |
| L131 | $\chi_{red}^{2}=1.05$ | $\chi_{red}^{2}=0.78$ | $\chi_{red}^{2}=0.68$  $k_{ex,bc}=140 s^{-1}$  $p_{c}=1.08\%$ |
| L192 | $\chi_{red}^{2}=3.47$ | $\chi_{red}^{2}=1.98$ | $\chi_{red}^{2}=2.03$  $k_{ex,bc}=350 s^{-1}$  $p_{c}=1.3\%$ |
| L198 | $\chi_{red}^{2}=1.51$ | $\chi_{red}^{2}=0.68$ | $\chi_{red}^{2}=0.65$  $k_{ex,bc}=1000 s^{-1}$  $p_{c}=1.2\%$ |
| L221 | $\chi_{red}^{2}=1.92$ | $\chi_{red}^{2}=1.57$ | $\chi_{red}^{2}=1.63$  $k_{ex,bc}=2600 s^{-1}$  $p_{c}=1.1\%$ |
| I237 | $\chi_{red}^{2}=4.20$ | $\chi_{red}^{2}=1.26$ | $\chi_{red}^{2}=1.24$  $k_{ex,bc}=230 s^{-1}$  $p_{c}=1.3\%$ |
