# Supplementary material for: Dynamic allostery in substrate binding by human thymidylate synthase
Source: eLife. 2022 Oct 6;11:e79915. doi: 10.7554/eLife.79915 (PMC9536839; doi:10.7554/eLife.79915)
Supplement: Supplementary file 2. — Probes involved in the concerted process are given the purple background. If a probe is fit to a 2-state model (A↔C), the parameters for the A↔B process are left blank. Cases where a 1H ∆ω is fixed at 0 are indicated by the ‘-‘. Parameter values are reported as median ±standard deviation of fit values from 200 Monte Carlo simulations of the data. For probes involved in the concerted process, the error in pb is the sum of the errors from the 2-state fit of the CEST data alone and from the global fit of the CPMG and CEST data with the slow process parameters fixed. Only the 13C ∆ω’s for the slow, concerted process have sign information from the CEST data; all other ∆ω’s should be interpreted only as the magnitude of the chemical shift difference. The background of each parameter value is color-coded based on the magnitude of the error relative to the parameter value, where blue indicates low error, yellow indicates medium error, and red indicates large error. In some cases, particularly for the populations, physical constraints on the parameter value (i.e. population cannot be less than 0) lead to highly skewed distributions of the parameter values in our Monte Carlo simulations. This can lead to nonsensical values when reported in our typical manner, for example the 0±6%pb given for L279 despite the fact that the population cannot be less than 0. In these cases, we have also listed (5% quantile, mode, 95% quantile) to provide greater insight into the distribution of values seen in the Monte Carlo simulations. For L269, marked with the asterisk, the CEST data was not included in the fit. Probes possessing Rex which weren’t analyzed include L67, V164, and V313. Refer to the legend of Supplementary file 7 for a description of the ‘met 1’ and ‘met 2’ labels. [file elife-79915-supp2.docx]

| Residue (met group) | $p_{a}$  $(\%)$ | $p_{b}$ | $p_{c}$ | $k_{ex,ab}$  $(s^{-1})$ | $k_{ex,ac}$ | $\Delta\omega_{C,ab}$  $(ppm)$ | $\Delta\omega_{H,ab}$ | $\Delta\omega_{C,ac}$ | $\Delta\omega_{H,ac}$ |
| --- | --- | --- | --- | --- | --- | --- | --- | --- | --- |
| L41 (met 2) | $99.86\pm0.03$ |  | $0.14\pm0.03$ |  | $1000\pm200$ |  |  | $1.5\pm0.3$ | - |
| V58 (met 1) | $85\pm9$ | $13\pm9$ | $1.1\pm0.2$ | $4\pm2$ | $800\pm100$ | $-0.70\pm0.02$ | - | $0.61\pm0.05$ | - |
| L73 (met 2) | $99.88\pm0.02$ |  | $0.12\pm0.02$ |  | $2400\pm300$ |  |  | $1.9\pm0.3$ | - |
| L73 (met 1) | $99.88\pm0.02$ |  | $0.12\pm0.02$ |  | $2400\pm300$ |  |  | $2.9\pm0.5$ | - |
| L74 (met 2) | $98.66\pm0.01$ | $1.34\pm$  0.01 |  | $241\pm7$ |  | $-0.75\pm0.01$ | - |  |  |
| V79 (met 1) | $98.52\pm0.02$ | $1.34\pm$  0.01 | $0.14\pm0.02$ | $241\pm7$ | $2900\pm500$ | $0.2\pm0.1$ | - | $3.1\pm0.3$ | - |
| L101 (met 2) | $98\pm1$ | $1.34\pm$  0.03 | $0\pm1$  (0.1,0.2,2.2) | $241\pm7$ | $7000\pm3000$ | $0.66\pm0.02$ | $0.23\pm0.02$ | $2\pm1$ | - |
| L101 (met 1) | $100\pm1$ |  | $0\pm1$  (0.1,0.2,2.2) |  | $7000\pm3000$ |  |  | $3\pm1$ | - |
| L118 (met 1) | $80\pm10$ |  | $20\pm10$ |  | $31000\pm9000$ |  |  | $1.0\pm0.4$ | - |
| L121 (met 1) | $95\pm2$ | $1.29\pm$  0.03 | $4\pm2$ | $241\pm7$ | $19000\pm7000$ | $0.39\pm0.02$ | - | $0.9\pm0.7$ | - |
| L131 (met 1) | $99\pm2$ | $1.34\pm$  0.04 | $0\pm2$  (0.1,0.1,1.0) | $241\pm7$ | $4000\pm2000$ | $0.68\pm0.03$ | $0.2\pm0.1$ | $1.9\pm0.8$ | - |
| L131 (met 2) | $100\pm2$ |  | $0\pm2$  (0.1,0.1,1.0) |  | $4000\pm2000$ |  |  | $4\pm1$ | - |
| L187 (met 1) | $98.66\pm0.01$ | $1.34\pm$  0.01 |  | $241\pm7$ |  | $0.417\pm0.005$ | $0.084\pm0.004$ |  |  |
| L192 (met 1) | $98.51\pm0.04$ | $1.34\pm$  0.01 | $0.16\pm0.04$ | $241\pm7$ | $2000\pm500$ | $-0.943\pm0.005$ | $0.224\pm0.006$ | $2.1\pm0.5$ | $0.28\pm0.04$ |
| L198 (met 2) | $98.42\pm0.04$ | $1.34\pm$  0.01 | $0.24\pm0.04$ | $241\pm7$ | $3300\pm700$ | $1.406\pm0.009$ | $0.23\pm0.01$ | $2.6\pm0.3$ | - |
| L212 (met 1) | $60\pm20$ |  | $40\pm20$ |  | $7000\pm1200$ |  |  | $0\pm1$  (0.2,0.2,3.8) | - |
| L221 (met 1) | $98.6\pm0.2$ | $1.34\pm$  0.02 | $0\pm0.2$  (0.03,0.04,0.16) | $241\pm7$ | $2000\pm2000$ | $0.863\pm0.005$ | $0.342\pm0.009$ | $3.1\pm0.7$ | - |
| I237 | $94.4\pm0.8$ | $1.28\pm$  0.02 | $4.3\pm0.9$ | $241\pm7$ | $12000\pm1000$ | $1.23\pm0.01$ | - | $3.3\pm0.7$ | - |
| L252 (met 2) | $99.86\pm0.05$ |  | $0.14\pm0.05$ |  | $1100\pm200$ |  |  | $1.4\pm0.5$ | - |
| L259 (met 1) | $74\pm5$ | $26\pm5$ | $0.11\pm0.01$ | $320\pm60$ | $860\pm70$ | $-0.06\pm0.01$ | - | $2.7\pm0.2$ | - |
| L269* (met 2) | $100\pm2$ |  | $1\pm2$  (0.2,0.3,4.6) |  | $700\pm200$ |  |  | $0.6\pm0.2$ | - |
| L279 (met 2) | $100\pm6$ |  | $0\pm6$  (0.1,0.1,0.3) |  | $1600\pm800$ |  |  | $3.2\pm0.7$ | - |
| V285 (met 2) | $100\pm20$ |  | $0\pm20$  (2,40,40) |  | $13000\pm7000$ |  |  | $3\pm2$ | - |
