# Supplementary material for: Dynamic allostery in substrate binding by human thymidylate synthase
Source: eLife. 2022 Oct 6;11:e79915. doi: 10.7554/eLife.79915 (PMC9536839; doi:10.7554/eLife.79915)
Supplement: Supplementary file 3. — All probes are fit to a 2-state model. Color-coding and values in parenthesis are as described in Supplementary file 2. L192 met 2, marked by the asterisk, is presumably the same methyl group analyzed in apo hTS given the similarity in the ∆ω’s, but assignment of this signal is complicated by a large chemical shift perturbation between apo and dUMP bound states. [file elife-79915-supp3.docx]

| Residue (met group) | $p_{b}$  $(\%)$ | $k_{ex}$  $(s^{-1})$ | $\Delta\omega_{C}$  $(ppm)$ | $\Delta\omega_{H}$ |
| --- | --- | --- | --- | --- |
| L41 (met 2) | $0\pm1$  (0.1,0.3,3.5) | $200\pm400$  (0,200,1300) | $2.2\pm0.4$ | - |
| L73 (met 1) | $1\pm2$  (0.2,0.4,5.1) | $0\pm1000$  (0,100,800) | $4.8\pm0.5$ | - |
| L101 (met 2) | $0\pm1$  (0.1,0.1,2.1) | $6000\pm4000$ | $2\pm1$ | - |
| L101 (met 1) | $0\pm1$  (0.1,0.1,2.1) | $6000\pm4000$ | $4.1\pm0.8$ | - |
| L118 (met 1) | $9\pm2$ | $44000\pm4000$ | $1.3\pm0.2$ | - |
| L121 (met 1) | $9\pm2$ | $45000\pm3000$ | $1.3\pm0.1$ | - |
| V158 (met 1) | $0.1\pm0.9$  (0.0,0.1,3.3) | $3000\pm3000$ | $4\pm1$ | - |
| L192 (met 2) | $0.1\pm0.3$  (0.1,0.1,0.4) | $1500\pm800$ | $3.9\pm0.6$ | $0.05\pm0.06$ |
| L192 (met 1)* | $0.1\pm0.3$  (0.1,0.1,0.4) | $1500\pm800$ | $2.4\pm0.5$ | $0.33\pm0.08$ |
| L198 (met 1) | $0.1\pm0.5$  (0.1,0.1,1.3) | $1800\pm700$ | $2.6\pm0.6$ | - |
| L198 (met 2) | $0.1\pm0.5$  (0.1,0.1,1.3) | $1800\pm700$ | $3.0\pm0.3$ | - |
| L221 (met 1) | $0.2\pm0.6$  (0.1,0.2,1.3) | $400\pm300$ | $3.8\pm0.3$ | - |
| L221 (met 2) | $0.2\pm0.6$  (0.1,0.2,1.3) | $400\pm300$ | $2.0\pm0.2$ | - |
| L232 (met 2) | $0\pm2$  (0.1,0.1,5.8) | $5000\pm9000$  (300,500,28100) | $4\pm1$ | - |
| L269 (met 2) | $0.2\pm0.6$  (0.1,0.2,2.0) | $200\pm600$  (0,200,800) | $1.4\pm0.5$ | - |
| L279 (met 2) | $0\pm1$  (0.1,0.2,4.2) | $400\pm600$  (0,400,1400) | $3.1\pm0.6$ | - |
| V285 (met 2) | $1.2\pm0.7$ | $9000\pm2000$ | $4.3\pm0.7$ | - |
