# Supplementary material for: Dynamic allostery in substrate binding by human thymidylate synthase
Source: eLife. 2022 Oct 6;11:e79915. doi: 10.7554/eLife.79915 (PMC9536839; doi:10.7554/eLife.79915)
Supplement: Supplementary file 4. — All probes are fit to a 2-state model. Color-coding and values in parenthesis are as described in Supplementary file 2. [file elife-79915-supp4.docx]

| Residue (met group) | $p_{b}$  $(\%)$ | $k_{ex}$  $(s^{-1})$ | $\Delta\omega_{C}$  $(ppm)$ | $\Delta\omega_{H}$ |
| --- | --- | --- | --- | --- |
| L41 (met 2) | $2\pm1$ | $40\pm500$  (20,20,970) | $1.9\pm0.3$ | - |
| L73 (met 2) | $0.1\pm0.2$  (0.1,0.1,0.6) | $4000\pm4000$ | $2.0\pm0.5$ | - |
| L73 (met 1) | $0.1\pm0.2$  (0.1,0.1,0.6) | $4000\pm4000$ | $4\pm1$ | - |
| V79 (met 1) | $0.2\pm0.5$  (0.1,0.1,1.1) | $1000\pm4000$  (100,200,10200) | $4.1\pm0.6$ | - |
| L101 (met 1) | $0.5\pm0.9$  (0.1,0.1,2.3) | $10000\pm5000$ | $2\pm1$ | - |
| L118 (met 1) | $15\pm5$ | $40000\pm10000$ | $0.6\pm0.3$ | - |
| L121 (met 1) | $5\pm1$ | $43000\pm3000$ | $1.2\pm0.1$ | - |
| L192 (met 1) | $0.9\pm0.7$ | $4000\pm2000$ | $0.7\pm0.5$ | $0.33\pm0.05$ |
| L198 (met 2) | $0.27\pm0.06$ | $4000\pm600$ | $2.5\pm0.3$ | - |
| L212 (met 1) | $0.2\pm0.3$  (0.1,0.1,1.0) | $8000\pm3000$ | $2.5\pm0.7$ | - |
| L221 (met 1) | $0.1\pm0.1$ | $1200\pm800$ | $2.8\pm0.2$ | - |
| L259 (met 1) | $0.5\pm0.2$ | $3800\pm200$ | $1.5\pm0.2$ | - |
| L269 (met 2) | $0.1\pm0.2$  (0.1,0.2,0.5) | $900\pm400$ | $1.7\pm0.2$ | - |
| L279 (met 1) | $1\pm1$ | $20000\pm7000$ | $1.8\pm0.4$ | - |
| L279 (met 2) | $1\pm1$ | $20000\pm7000$ | $1.7\pm0.4$ | - |
| V285 (met 2) | $0.9\pm0.5$ | $7000\pm2000$ | $4.8\pm0.6$ | - |
