# Supplementary material for: Dynamic allostery in substrate binding by human thymidylate synthase
Source: eLife. 2022 Oct 6;11:e79915. doi: 10.7554/eLife.79915 (PMC9536839; doi:10.7554/eLife.79915)
Supplement: Supplementary file 5. [file elife-79915-supp5.docx]

| Experiment | # indirect complex points | Indirect spectral width (ppm) | Scans/FID | Interscan delay (s) | # planes | Relaxation time (ms) |
| --- | --- | --- | --- | --- | --- | --- |
| ^1^H-^15^N RDCs (apo/dUMP) | 125 | 35 | 32 | 2 | - | - |
| MQ CPMG (apo/dUMP, 850/600 MHz) | 60 | 9 | 8 | 1.5 | 27 (2 duplicates, 50 – 2000 Hz $\upsilon_{cpmg}$) | 40 |
| ^13^C SQ CPMG (apo/dUMP, 850 MHz) | 100 | 18 | 8 | 2 | 20 (2 duplicates, 100 – 2000 Hz $\upsilon_{cpmg}$) | 20 |
| ^13^C CEST (apo 600 MHz, 41/25 Hz spin lock) | 100 | 18 | 4 | 2 | 71 (40 Hz steps)/113 (25 Hz steps) | 400 |
| sPRE | 88 | 9 | 16 | 1.2 | - | - |
| ^2^H R_2_ (apo/dUMP/TMP, 600 MHz) | 100 | 18 | 24 | 2 | 7 | 0 - 2 |
| ^2^H R_2_ (Δ25 apo, 600 MHz) | 100 | 18 | 24 | 2 | 9 (2 duplicates) | 0 - 3 |
| ^2^H R_2_ (Δ25 dUMP, 600 MHz) | 130 | 18 | 16 | 2 | 9 (2 duplicates) | 0 - 3 |
| ^15^N $\boldsymbol{T}_{\boldsymbol{1}}$ (apo, 600 MHz) | 120 | 35 | 8 | 3.5 | 11 (2 duplicates) | 0 - 3600 |
| ^15^N $\boldsymbol{T}_{\boldsymbol{1}}$ (apo, 500 MHz) | 150 | 35 | 8 | 3.5 | 11 (2 duplicates) | 0 - 3600 |
| ^15^N $\boldsymbol{T}_{\boldsymbol{1}\boldsymbol{\rho}}$ (apo, 600 MHz) | 120 | 35 | 8 | 3.5 | 11 (2 duplicates) | 0 - 70 |
| ^15^N $\boldsymbol{T}_{\boldsymbol{1}\boldsymbol{\rho}}$ (apo, 500 MHz) | 150 | 35 | 16 | 3.5 | 11 (2 duplicates) | 0 - 70 |
| Heteronuclear NOE (apo, 600 MHz) | 120 | 35 | 32 | 12/1 + 11 saturation | - | - |
| Heteronuclear NOE (apo, 500 MHz) | 140 | 35 | 24 | 12/1 + 11 saturation | - | - |
| ^15^N $\boldsymbol{T}_{\boldsymbol{1}}$ (dUMP, 600 MHz) | 120 | 35 | 8 | 3.5 | 11 (2 duplicates) | 0 - 3600 |
| ^15^N $\boldsymbol{T}_{\boldsymbol{1}}$ (dUMP, 500 MHz) | 120 | 35 | 8 | 3.5 | 9 (2 duplicates) | 0 - 2800 |
| ^15^N $\boldsymbol{T}_{\boldsymbol{1}\boldsymbol{\rho}}$ (dUMP, 600 MHz) | 120 | 35 | 16 | 3.5 | 11 (2 duplicates) | 0 - 70 |
| ^15^N $\boldsymbol{T}_{\boldsymbol{1}\boldsymbol{\rho}}$ (dUMP, 500 MHz) | 120 | 35 | 16 | 3.5 | 10 (2 duplicates) | 0 - 70 |
| Heteronuclear NOE (dUMP, 600 MHz) | 120 | 35 | 28 | 12/1 + 11 saturation | - | - |
| ^15^N $\boldsymbol{T}_{\boldsymbol{1}}$ (TMP, 600 MHz) | 130 | 35 | 8 | 3.5 | 9 (2 duplicates) | 0 - 3600 |
| ^15^N $\boldsymbol{T}_{\boldsymbol{1}\boldsymbol{\rho}}$ (TMP, 600 MHz) | 140 | 35 | 8 | 3.5 | 11 (2 duplicates) | 0 - 50 |
| Heteronuclear NOE (TMP, 600 MHz) | 130 | 35 | 32 | 12/1 + 11 saturation | - | - |
| ^15^N $\boldsymbol{T}_{\boldsymbol{1}}$ (Δ25 apo, 600 MHz) | 120 | 35 | 8 | 3.5 | 11 (2 duplicates) | 0 - 3600 |
| ^15^N $\boldsymbol{T}_{\boldsymbol{1}\boldsymbol{\rho}}$ (Δ25 apo, 600 MHz) | 120 | 35 | 8 | 3.5 | 11 (2 duplicates) | 0 - 50 |
| Heteronuclear NOE (Δ25 apo, 600 MHz) | 92 | 35 | 32 | 12/1 + 11 saturation | - | - |
| ^15^N $\boldsymbol{T}_{\boldsymbol{1}}$ (Δ25 dUMP, 600 MHz) | 120 | 30 | 8 | 3.5 | 11 (2 duplicates) | 0 - 3600 |
| ^15^N $\boldsymbol{T}_{\boldsymbol{1}\boldsymbol{\rho}}$ (Δ25 dUMP, 600 MHz) | 120 | 30 | 16 | 3.5 | 11 (2 duplicates) | 0 - 50 |
| ^1^H-^13^C HMQC (full length apo) | 150 | 25 | 8 | 1.5 | - | - |
| ^1^H-^13^C HMQC (Δ25 apo) | 88 | 9 | 8 | 1.2 | - | - |
| 1D selective CEST (apo, 850 MHz) | - | - | 64 | 2 | 50 (25 Hz steps) | 400 |
| MQ CPMG (Δ25 apo, 850/600 MHz) | 60 | 9 | 8 | 1.5 | 27 (2 duplicates, 50 – 2000 Hz $\upsilon_{cpmg}$) | 40 |
| SQ CPMG (Δ25 apo, 850 MHz) | 60 | 9 | 12 | 1.5 | 20 (2 duplicates, 100 – 2000 Hz $\upsilon_{cpmg}$) | 20 |
| ^1^H CPMG 2-plane (apo, 850 MHz) | 100 | 18 | 4 | 2 | 2 (50 and 2000 Hz $\upsilon_{cpmg}$) | 20 |
