# Supplementary material for: Dynamic allostery in substrate binding by human thymidylate synthase
Source: eLife. 2022 Oct 6;11:e79915. doi: 10.7554/eLife.79915 (PMC9536839; doi:10.7554/eLife.79915)
Supplement: Supplementary file 6. — Rotational correlation times for various hTS-bound states determined by 15N relaxation (Source data 1–19). [file elife-79915-supp6.docx]

| **hTS bound state** | $\boldsymbol{\tau}_{\boldsymbol{c,5\%}\boldsymbol{D}_{\boldsymbol{2}}\boldsymbol{O}}\boldsymbol{(ns)}$ |
| --- | --- |
| apo | $44.71\pm0.04$ |
| dUMP | $43.93\pm0.05$ |
| TMP | $44.85\pm0.06$ |
| Δ25 apo | $38.50\pm0.04$ |
| Δ25 dUMP | $36.28\pm0.05$ |
