# Supplementary material for: Dynamic allostery in substrate binding by human thymidylate synthase
Source: eLife. 2022 Oct 6;11:e79915. doi: 10.7554/eLife.79915 (PMC9536839; doi:10.7554/eLife.79915)
Supplement: Supplementary file 7. — For LV methyls, the labels ‘met1’ and ‘met2’ are given, where ‘met1’ has the larger 13C chemical shift, as stereospecific assignments have not been made. For all other states, the ‘met1’ and ‘met2’ labels are given based on chemical shift similarity to the apo state. The only exception is L192, where ∆ω’s obtained from our dispersion fits guided the assignment. [file elife-79915-supp7.docx]

| Residue (met group) | S^2^axis | Error |
| --- | --- | --- |
| 3met1 | 0.048 | 0.003 |
| 3met2 | 0.043 | 0.002 |
| 31met1 | 0.65 | 0.02 |
| 34met1 | 0.9 | 0.05 |
| 37 | 0.4 | 0.02 |
| 40 | 0.88 | 0.03 |
| 41met1 | 0.9 | 0.04 |
| 41met2 | 0.79 | 0.03 |
| 56met1 | 0.74 | 0.03 |
| 58met2 | 0.51 | 0.03 |
| 67met1 | 1 | 0.05 |
| 67met2 | 0.84 | 0.04 |
| 73met2 | 0.74 | 0.04 |
| 85met1 | 0.77 | 0.03 |
| 85met2 | 0.78 | 0.03 |
| 88met1 | 0.61 | 0.03 |
| 88met2 | 0.7 | 0.04 |
| 89met2 | 0.83 | 0.04 |
| 89met1 | 0.59 | 0.02 |
| 92 | 0.92 | 0.02 |
| 101met2 | 0.84 | 0.06 |
| 121met1 | 0.55 | 0.02 |
| 158met2 | 0.81 | 0.05 |
| 161met1 | 0.84 | 0.06 |
| 165 | 0.66 | 0.02 |
| 168 | 0.9 | 0.03 |
| 178 | 0.4 | 0.02 |
| 187met1 | 0.25 | 0.01 |
| 187met2 | 0.29 | 0.02 |
| 189met1 | 0.55 | 0.02 |
| 192met1 | 0.18 | 0.02 |
| 198met2 | 0.35 | 0.04 |
| 203met2 | 1.02 | 0.06 |
| 208met1 | 0.89 | 0.05 |
| 221met2 | 0.32 | 0.02 |
| 232met2 | 0.93 | 0.04 |
| 232met1 | 0.91 | 0.05 |
| 233met1 | 1.04 | 0.04 |
| 237 | 0.48 | 0.04 |
| 240 | 0.73 | 0.02 |
| 243met1 | 0.92 | 0.05 |
| 249met1 | 0.43 | 0.02 |
| 257 | 0.88 | 0.02 |
| 259met1 | 0.49 | 0.02 |
| 262 | 0.58 | 0.01 |
| 267 | 0.398 | 0.005 |
| 269met1 | 0.51 | 0.02 |
| 279met1 | 0.74 | 0.03 |
| 279met2 | 0.65 | 0.04 |
| 281 | 0.75 | 0.02 |
| 288 | 0.327 | 0.008 |
| 298 | 0.46 | 0.01 |
| 307 | 0.53 | 0.03 |
| 313met1 | 0.052 | 0.003 |
| 313met2 | 0.055 | 0.004 |
