# Supplementary material for: Dynamic allostery in substrate binding by human thymidylate synthase
Source: eLife. 2022 Oct 6;11:e79915. doi: 10.7554/eLife.79915 (PMC9536839; doi:10.7554/eLife.79915)
Supplement: Supplementary file 9. [file elife-79915-supp9.docx]

| Residue (met group) | S^2^axis | Error |
| --- | --- | --- |
| 3met1 | 0.044 | 0.003 |
| 3met2 | 0.085 | 0.003 |
| 31met1 | 0.68 | 0.02 |
| 31met2 | 0.6 | 0.02 |
| 34met1 | 0.82 | 0.05 |
| 37 | 0.44 | 0.02 |
| 40 | 0.92 | 0.03 |
| 41met1 | 0.78 | 0.03 |
| 41met2 | 0.73 | 0.03 |
| 56met1 | 0.67 | 0.02 |
| 58met2 | 0.53 | 0.03 |
| 67met1 | 0.91 | 0.04 |
| 67met2 | 0.68 | 0.04 |
| 73met2 | 0.77 | 0.04 |
| 73met1 | 0.74 | 0.05 |
| 74met2 | 0.75 | 0.06 |
| 85met2 | 0.77 | 0.03 |
| 88met1 | 0.49 | 0.03 |
| 88met2 | 0.76 | 0.04 |
| 89met2 | 0.97 | 0.05 |
| 89met1 | 0.55 | 0.02 |
| 92 | 0.85 | 0.02 |
| 101met2 | 0.89 | 0.05 |
| 108 | 0.64 | 0.02 |
| 121met1 | 0.69 | 0.03 |
| 158met2 | 0.78 | 0.04 |
| 164met2 | 0.96 | 0.07 |
| 165 | 0.59 | 0.01 |
| 168 | 0.89 | 0.03 |
| 178 | 0.45 | 0.02 |
| 187met1 | 0.23 | 0.01 |
| 187met2 | 0.26 | 0.01 |
| 189met1 | 0.58 | 0.02 |
| 192met1 | 0.41 | 0.03 |
| 198met1 | 0.37 | 0.02 |
| 198met2 | 0.39 | 0.03 |
| 203met1 | 0.89 | 0.06 |
| 203met2 | 0.99 | 0.06 |
| 208met1 | 1.1 | 0.07 |
| 221met1 | 0.44 | 0.02 |
| 221met2 | 0.46 | 0.02 |
| 232met2 | 0.88 | 0.04 |
| 232met1 | 0.8 | 0.05 |
| 233met1 | 1.04 | 0.05 |
| 237 | 0.39 | 0.04 |
| 240 | 0.73 | 0.02 |
| 243met2 | 0.91 | 0.06 |
| 243met1 | 0.94 | 0.06 |
| 257 | 0.83 | 0.02 |
| 259met2 | 0.56 | 0.02 |
| 262 | 0.63 | 0.01 |
| 267 | 0.405 | 0.005 |
| 269met1 | 0.46 | 0.01 |
| 269met2 | 0.37 | 0.02 |
| 279met1 | 0.74 | 0.03 |
| 279met2 | 0.57 | 0.05 |
| 281 | 0.74 | 0.02 |
| 282met1 | 0.57 | 0.02 |
| 288 | 0.318 | 0.008 |
| 298 | 0.46 | 0.01 |
| 307 | 0.49 | 0.03 |
| 313met1 | 0.061 | 0.003 |
| 313met2 | 0.062 | 0.004 |
