# Supplementary material for: Dynamic allostery in substrate binding by human thymidylate synthase
Source: eLife. 2022 Oct 6;11:e79915. doi: 10.7554/eLife.79915 (PMC9536839; doi:10.7554/eLife.79915)
Supplement: Supplementary file 10. [file elife-79915-supp10.docx]

| Residue (met group) | S^2^axis | Error |
| --- | --- | --- |
| 31met1 | 0.66 | 0.01 |
| 34met1 | 0.85 | 0.02 |
| 34met2 | 0.83 | 0.02 |
| 37 | 0.395 | 0.007 |
| 40 | 0.85 | 0.02 |
| 41met1 | 0.87 | 0.02 |
| 41met2 | 0.76 | 0.02 |
| 45met1 | 0.404 | 0.004 |
| 45met2 | 0.359 | 0.005 |
| 56met1 | 0.72 | 0.01 |
| 58met2 | 0.571 | 0.007 |
| 67met1 | 0.89 | 0.02 |
| 67met2 | 0.92 | 0.02 |
| 73met2 | 0.8 | 0.02 |
| 74met2 | 0.81 | 0.02 |
| 74met1 | 1.02 | 0.04 |
| 79met1 | 0.92 | 0.03 |
| 84met1 | 0.88 | 0.03 |
| 84met2 | 1 | 0.04 |
| 85met1 | 0.82 | 0.02 |
| 85met2 | 0.81 | 0.02 |
| 88met1 | 0.57 | 0.01 |
| 88met2 | 0.93 | 0.03 |
| 89met2 | 0.95 | 0.02 |
| 89met1 | 0.59 | 0.01 |
| 92 | 0.84 | 0.02 |
| 101met2 | 0.83 | 0.02 |
| 101met1 | 0.87 | 0.02 |
| 106met2 | 0.94 | 0.02 |
| 118met2 | 0.96 | 0.03 |
| 118met1 | 0.99 | 0.03 |
| 121met1 | 0.593 | 0.01 |
| 131met2 | 0.93 | 0.02 |
| 131met1 | 0.98 | 0.03 |
| 134met2 | 0.96 | 0.03 |
| 158met2 | 0.88 | 0.02 |
| 161met1 | 0.91 | 0.03 |
| 164met2 | 0.98 | 0.03 |
| 164met1 | 0.91 | 0.04 |
| 165 | 0.69 | 0.01 |
| 168 | 0.97 | 0.03 |
| 177 | 0.95 | 0.03 |
| 178 | 0.473 | 0.007 |
| 187met1 | 0.274 | 0.003 |
| 187met2 | 0.259 | 0.004 |
| 189met1 | 0.568 | 0.007 |
| 192met1 | 0.196 | 0.004 |
| 198met2 | 0.355 | 0.007 |
| 203met2 | 1.02 | 0.02 |
| 204met2 | 0.74 | 0.01 |
| 208met1 | 0.99 | 0.03 |
| 212met2 | 0.95 | 0.04 |
| 221met1 | 0.3 | 0.004 |
| 221met2 | 0.327 | 0.004 |
| 223met2 | 1.14 | 0.04 |
| 223met1 | 0.96 | 0.03 |
| 232met2 | 0.9 | 0.03 |
| 232met1 | 0.99 | 0.03 |
| 233met1 | 0.95 | 0.02 |
| 237 | 0.54 | 0.03 |
| 240 | 0.73 | 0.02 |
| 243met2 | 1.12 | 0.06 |
| 243met1 | 1.03 | 0.03 |
| 249met1 | 0.392 | 0.007 |
| 252met2 | 0.7 | 0.01 |
| 252met1 | 0.77 | 0.02 |
| 257 | 0.85 | 0.02 |
| 259met1 | 0.536 | 0.008 |
| 262 | 0.654 | 0.01 |
| 265met1 | 0.89 | 0.02 |
| 265met2 | 0.7 | 0.02 |
| 267 | 0.413 | 0.006 |
| 269met1 | 0.498 | 0.007 |
| 269met2 | 0.427 | 0.006 |
| 279met1 | 0.77 | 0.02 |
| 279met2 | 0.69 | 0.02 |
| 281 | 0.77 | 0.02 |
| 285met1 | 0.7 | 0.02 |
| 288 | 0.325 | 0.006 |
| 298 | 0.496 | 0.007 |
| 307 | 0.54 | 0.01 |
| 313met1 | 0.074 | 0.001 |
| 313met2 | 0.064 | 0.002 |
